# Supplementary material for: An open-source framework for large-scale, flexible evaluation of biomedical text mining systems
Source: J Biomed Discov Collab. 2008 Jan 29;3:1. doi: 10.1186/1747-5333-3-1 (PMC2276192; doi:10.1186/1747-5333-3-1)
Supplement: Additional file 1 — Evaluation platform source code. The additional file contains the software evaluation framework discussed in this paper. The most current release can be downloaded from SourceForge.net [1]. See the accompanying README file for instructions on installation and use. Also included in the distribution is a collection of UIMA wrappers for some commonly used BioNLP tools and annotated corpora. [file 1747-5333-3-1-S1.zip › bionlp_uima_repository-1.0.1/docs/api/src-html/edu/uchsc/ccp/util/nlp/mention/Mention.html]

```
001    /*
002     *
003     * The Original Code is UCHSC Utilities.
004     *
005     * The Initial Developer of the Original Code is University of Colorado.  
006     * Copyright (C) 2005.  All Rights Reserved.
007     *
008     * UCHSC Utilities was developed by the Center for Computational Pharmacology
009     * (http://compbio.uchsc.edu) at the University of Colorado Health 
010     *  Sciences Center School of Medicine with support from the National 
011     *  Library of Medicine.  
012     *
013     *
014     * Contributor(s):
015     *   William A. Baumgartner <william.baumgartner@uchsc.edu> (Original Author)
016     *   
017     * Created: November 2005
018     * Dependencies:
019     * TODO: 
020     * Changes:
021     */
022    
023    package edu.uchsc.ccp.util.nlp.mention;
024    
025    
026    public class Mention implements Comparable {
027    
028        private String mentionName;
029    
030        /**
031         * 
032         */
033        @Deprecated
034        public Mention() {
035            super();
036            mentionName = "";
037        }
038    
039        public Mention(String mentionName) {
040            super();
041            this.mentionName = mentionName;
042        }
043    
044        public String getMentionName() {
045            return mentionName;
046        }
047    
048        @Deprecated
049        public void setMentionName(String mentionName) {
050            this.mentionName = mentionName;
051        }
052    
053        
054        public String getSingleLineRepresentation() {
055            throw new UnsupportedOperationException("getSingleLineRepresenation() must be implemented by subclasses of the Mention class.");
056        }
057        
058        public String getDocumentLevelSingleLineRepresentation() {
059            throw new UnsupportedOperationException("getDocumentLevelSingleLineRepresenation() must be implemented by subclasses of the Mention class.");
060        }
061        
062        /**
063         * Must be implemented by subclasses of Mention
064         * 
065         * @param o
066         * @return
067         */
068        public int compareTo(Object o) {
069            throw new UnsupportedOperationException("compareTo() must be implemented by subclasses of the Mention class.");
070        }
071    
072    
073    }
```
